# Phytochemical Evaluation of *Lepidium meyenii*, *Trigonella foenum-graecum*, *Spirulina platensis*, and *Tribulus arabica*, and Their Potential Effect on Monosodium Glutamate Induced Male Reproductive Dysfunction in Adult Wistar Rats

**DOI:** 10.3390/antiox13080939

**Published:** 2024-08-02

**Authors:** Naglaa Gamil Shehab, Temidayo S. Omolaoye, Stefan S. Du Plessis, Surendra Singh Rawat, Nerissa Naidoo, Kholoud Y. Abushawish, Ayat Ahmed, Baraa Alaa, Heba Ihsan, Manar Abdelhalim, Mariam Ayman, Eslam El Nebrisi

**Affiliations:** 1 Pharmaceutical Sciences Department, Dubai Pharmacy College for Girls, Dubai 19099, United Arab Emirates; khuloody@dpc.edu; 2 Pharmacognosy Department, Faculty of Pharmacy, Cairo University, Giza 11562, Egypt; 3 College of Medicine, Mohammed Bin Rashid University of Medicine and Health Sciences, Dubai Health, Dubai P.O. Box 505055, United Arab Emirates; temidayo.omolaoye@mbru.ac.ae (T.S.O.); stefan.duplessis@dubaihealth.ae (S.S.D.P.); surendrasingh.rawat@dubaihealth.ae (S.S.R.); nerissa.naidoo@mbru.ac.ae (N.N.); 4 Dubai Pharmacy College for Girls, Dubai 19099, United Arab Emirates; aya20190205@dpc.edu (A.A.); bam20190206@dpc.edu (B.A.); hlb20190244@dpc.edu (H.I.); mam20190225@dpc.edu (M.A.); maa20190228@dpc.edu (M.A.); 5 Department of Biomedical Sciences, Dubai Medical College for Girls, Dubai 20170, United Arab Emirates

**Keywords:** monosodium glutamate, male infertility, *Spirulina platensis*, *Lepidium meyenii*, *Tribulus arabica*, *Trigonella foenum-graecum*, sperm parameters, antioxidants

## Abstract

Monosodium glutamate (MSG), a sodium salt derived from glutamic acid, is widely used in commercial food products to improve taste, quality, and preservation. However, its consumption may have detrimental effects on male reproductive function. Nevertheless, plant extracts, such as *Lepidium meyenii* (Maca), *Trigonella foenum-graecum* (Fenugreek), *Spirulina platensis* (Spirulina), and *Tribulus arabica* (Tribulus), may ameliorate these adverse effects. To this effect, the phytochemical properties of *Lepidium meyenii*, *Trigonella foenum-graecum*, *Spirulina platensis*, and *Tribulus arabica* were assessed, and their potential impact on MSG-induced impairment of reproductive parameters was examined. The phytochemical composition (steroids, terpenes, phenols, flavonoids) of the plants was profiled through spectrophotometry and the antioxidant activity was assessed using the 1,1-diphenyl-2-picrylhydrazyl (DPPH) radical scavenging assay. Thirty-six male Wistar rats were divided into six groups at random: a control group receiving distilled water, and five experimental groups (MSG, Maca, Fenugreek, Spirulina, and Tribulus) receiving 900 mg/kg/day of MSG dissolved in water for 45 days. Subsequently, the animals in the experimental groups were administered 500 mg/kg/day of the respective plant extract via oral gavage for an additional 35 days, while the MSG group continued to receive water only. Following the treatment period, the animals were sacrificed, and their reproductive tract organs were collected, weighed, and subjected to further analysis. Phytochemical analysis revealed the presence of diverse bioactive elements in the plant extracts, including phenolic and flavonoid compounds. Exposure to MSG negatively impacted total and progressive sperm motility, which was ameliorated by *Lepidium meyenii* treatment. Sperm morphology showed no significant differences among groups. Treatment of the phytochemical agents diminished histomorphometric alternations of the testicular length, germinal epithelium height, and number of cells in seminiferous tubules, which were caused by the initial administration of MSG. Testosterone and LH levels were reduced in the MSG group but improved in extract-treated groups. The study suggests *Lepidium meyenii* as a potential remedy for reproductive dysfunction. However, further investigation into its mechanisms and human safety and efficacy is warranted.

## 1. Introduction

Infertility is defined as the inability to achieve pregnancy after 12 months of regular, unprotected sexual contact [1]. It is now considered a global health issue due to its increasing prevalence in couples in their reproductive years, especially those in developing nations [2]. According to a recent prolonged systematic meta-analysis study over 30 years (2022), the infertility prevalence estimates reported by male respondents were generally lower than those reported by female respondents, suggesting potential differences in the experience and reporting of infertility between sexes [3]. Male infertility is influenced by diverse factors including diet [4], stress [5], smoking [6], excessive exercise [7,8], genetic aberration [9], chemical exposure that disrupts the hormonal system [10,11], heat [12], genital inflammation [13], food additives such as monosodium glutamate (MSG) [14], and several other factors [15]. MSG, derived from the sodium salt of glutamic acid, is commonly used as a seasoning and flavor enhancer in food products to enhance taste, quality, and shelf-life. Although it is generally regarded as safe, in terms of safety, it remains controversial for use in feed additives [16]. Due to the efficient metabolism of glutamate by enterocytes, only a minute portion of ingested MSG is absorbed into the bloodstream, increasing plasma glutamate levels transiently. However, when consumed excessively, it may pose a danger to health. Studies have highlighted that the excessive consumption of MSG is associated with adverse effects such as headaches, numbness, flushing generalized weakness, dizziness, and muscle pain [16]. In recent years, the detrimental impact of MSG on the male reproductive system and its functions has been extensively detailed by Kayode et al. [17]. Some of the identified mechanisms and consequent effects of MSG include spermatogenic alteration resulting in oligospermia, teratozoospermia, and necrospermia. Oxidative damage, histological alteration (hemorrhage, distorted germ and Sertoli cells), as well as gonadotropin imbalance (reduced testosterone, luteinizing hormone (LH), and follicle-stimulating hormone (FSH) concentrations) have likewise been reported [18]. Although these adverse effects can be managed with conventional therapy such as hormonal treatment or surgical interventions, the use of natural compounds with stimulatory effects on male reproductive health may also serve as an alternative treatment strategy.

*Spirulina platensis* (Spirulina), a type of photosynthetic cyanobacteria, commonly known as blue-green algae, belongs to the Oscillatoriaceae family. It is multicellular, filamentous, and has a distinctive spiral shape. It is classified under the Cyanophyceae class. *Spirulina platensis* is cultivated globally and serves as a primary dietary supplement for numerous individuals [19]. Likewise, it is utilized in aquaculture and poultry farming as a feed supplement [20]. It has a wide range of prophylactic and curative nutrients, including vitamins, minerals, proteins, linolenic acid, carotene, and undiscovered bioactive compounds [21]. Along with its nutritional benefits, *S. platensis* also possesses antibacterial, antifungal, antiviral, anticancer, anti-inflammatory, and antioxidant properties [22].

Studies have reported its protective and ameliorative potential on spermatogenesis in animals exposed to cadmium [23] and silver particles [24]. Similarly, supplementation of *Spirulina* to semen extender has been shown to influence the quality and antioxidant parameters of chilled or cryopreserved Arabian stallion spermatozoa [25].

*Lepidium meyenii* (Maca), belongs to the family Brassicaceae, growing widespread in various South American nations. It is rich in many chemical constituents with bioactivity including glucosinolates, macamides, macaenes, thiohydantoins, and alkaloids. For centuries, the Andes have used Maca as an adaptogenic plant to treat anaemia, and for balancing of female hormones. More recently, Maca has been introduced to Japan, Europe, and the US, and it is now being used more frequently throughout the world [26].

*Tribulus arabica* is an annual herbaceous plant in the caltrop family (Zygophyllaceae), that grows all over the globe. The major active ingredients of *T. arabica* are furostanol saponins known as protodioscins. Both Indian and Chinese traditional medicine have used it to treat a variety of diseases [27].

Fenugreek, scientifically known as *Trigonella foenum-graecum*, is an annual plant belonging to the Fabaceae family. A single leaf consists of three small obovate or oblong leaflets. This plant is cultivated globally as a semiarid crop. The seeds and leaves of this plant have been used as a culinary ingredient in the Indian subcontinent for a long time and are a popular addition to dishes in that region. The seeds are composed of protein, starch, sugar, mucilage, minerals, risky oil, constant oil, nutrients, and enzymes.

While Fenugreek is commonly offered as a nutritional supplement and utilized in conventional medicine [28], several studies have indicated its potential significant adverse effects, including allergic reactions [29]. Therefore, there is a lack of medical evidence supporting the healing properties of Fenugreek.

While the medicinal potential of Maca, Fenugreek (in low quantity), *Spirulina* and *Tribulus* have been reported in diverse pathophysiological conditions, studies investigating their effect on MSG-induced reproductive function impairment are limited. Additionally, data on the phytochemical composition of these plant extracts are lacking. Thus, the present study aimed to examine the phytochemical constituents of *Lepidium meyenii*, *Trigonella foenum-graecum*, *Spirulina platensis* and *Tribulus arabica*, extracts and to assess their potential effect on monosodium glutamate-induced male reproductive dysfunction in adult Wistar rats

## 2. Materials and Methods

### 2.1. Chemicals

Ethanol and Methanol were purchased from Honeywell Specialty Chemicals, Wunstorfer Strasse, Seelze, Germany. While Folin- Ciocalteu reagent, gallic acid, NaNO_2_, aluminum chloride, sodium hydroxide, monosodium glutamate (MSG) and carboxymethyl cellulose were purchased from Merck, Darmstadt, Germany. Follicle-stimulating hormone (FSH), luteinizing hormone (LH), testosterone and total antioxidant capacity kits were purchased from Elabscience Biotechnology Inc., Houston, TX, USA.

### 2.2. Phytochemical Evaluation of the Plant’s Constituents

#### 2.2.1. Plant Materials and Extraction

*Spirulina platensis*, *Lepidium meyenii*, and *Trigonella foenum-graecum* were purchased from the public market in September 2022, in Dubai, UAE, whereas *Tribulus arabica* was collected from Muhaisnah 1 desert, Dubai, UAE in September 2022. All the plants were identified by Prof. Naglaa Gamil Shehab, affiliated with the Pharmaceutical Sciences Department, Dubai Pharmacy College for Girls, Dubai, UAE and the Pharmacognosy Department, Faculty of Pharmacy, Cairo University (Cairo, Egypt).

Voucher specimens were kept at the Herbarium of the Pharmaceutical Sciences Department (# 6-9-22). One kilogram from each plant under investigation was powdered and then macerated in 70% alcohol (2 L each) at room temperature using ultrasound for one week. The extracts were filtered and evaporated under reduced pressure using a rotary evaporator at 50 °C. The remaining aqueous extracts were freeze-dried at −46 °C, under 1 pa, using a lyophilizer (BK-FD10P, BIOBASE, Jinan, China). The extracts were stored in the fridge at 4 °C for further analysis.

#### 2.2.2. Determination of Phenolic and Flavonoid Contents

The total phenolic contents were determined using the Folin-Ciocalteu reagent (Merck, Darmstadt, Germany) following a method described by Singleton and Rossi and further modified by Shehab et al. [30]. The quantification was reported in milligrams per gram gallic acid equivalent, based on the dry weight of the plant material. A calibration curve was constructed using serial dilutions of gallic acid (10, 20, 30, 40, and 50 μg/mL). The tested samples and standards were individually mixed with one ml of Folin-Ciocalteu reagent and nine mL of water in a volumetric flask, and the resulting mixture was carefully vortexed. After five minutes, 10 mL of 7% sodium carbonate was added, and the reaction mixture was kept at room temperature for an additional 90 min. Finally, the absorbance was measured at 750 nm against a reagent blank.

The quantification of flavonoids in the extracts was performed using the aluminum chloride method with quercetin as a reference standard, following the procedure described by Shehab et al. [30]. Spectrophotometry was employed to measure the total flavonoid content. To accomplish this, 0.1 mL of each plant extract was mixed with 0.3 mL of distilled water and 70% alcohol. The resulting mixture, exhibiting a yellow color, was then subjected to absorbance measurement at 510 nm.

#### 2.2.3. Quantitative Determination of the Phenolic Acids and Flavonoids Constituents by RP-HPLC

Quantitative determination of the phenolic acids and flavonoids constituents were performed by RP-HPLC equipment (Hipersep Prochrom version 450 Agilent Series 1100) (Agilent, Santa Clara, CA, USA). The apparatus consists of an auto-sampling injector, a solvent degasser, two LC pumps (series 1100), ChemStation B04.03, and a UV-Vis detector set at 250 nm for phenolic acids and 360 nm for flavonoids. The analysis was conducted using a C18 column (125 mm × 4.60 mm, 5 µm particle size). Phenolic acids were separated using a gradient mobile phase of two solvents: Solvent A (Methanol) and Solvent B (Acetic acid in water, 1:25). The gradient program started with 100% B for the first 3 min, followed by 50% A for the next 5 min, then increased to 80% A for 2 min, and finally returned to 50% A for the last 5 min. The identification of the constituents was carried out at wavelength 250 nm. On the other hand, flavonoid constituents were separated by employing the mobile phase of two solvents: acetonitrile (A) and 0.2% (*v*/*v*) aqueous formic acid (B) with an isocratic elution (70:30) program. The solvent flow rate was 1 mL/min, and separation was performed at 25 °C. The injection volumes were 25 μL. The identification of the constituents was measured at wavelength 360 nm. Individual components were identified by comparing the retention times of unknown peaks to those of reference standards. Samples analyses were performed in triplicate [31,32].

#### 2.2.4. Evaluation of the Antioxidant Activity of the Plant Extract

The 2,2-Diphenyl-1-picrylhydrazyl (DPPH) radical scavenging assay was utilized. To measure the free radical-scavenging abilities of the extracts from the four plants prepared in 70% alcohol, the stable DPPH radical was used through hydrogen donation or radical scavenging. The assay was conducted in a 96-well microtiter plate, following a modified version of a previously described method. In each well, 100 μL of both the sample and standard solution were mixed with 100 μL of a 0.1 mM ethanolic DPPH solution. Subsequently, the reaction mixtures were vigorously agitated and incubated in a light-free environment at a temperature of 37 °C for a duration of 30 min. The absorbance at a wavelength of 517 nm was determined using a UV-vis microplate reader. The percentage inhibition of the DPPH radical by the samples was then calculated using the following formula:

% inhibition = [A0 − (A1 − A2)]/A0 × 100%.

where A0 represents the absorbance of the control, A1 denotes the absorbance while the sample is present, and A2 is the sample’s absorbance under the same circumstances as A1 but using ethanol rather than DPPH solution.

### 2.3. Ethics and Animal Care

Ethical approval was obtained from the Dubai Pharmacy College Ethics Committee (REC/UG/30/06/2022). The treatment of animals followed the international guidelines for the care and use of laboratory animals [33]. Thirty-six healthy male Wistar rats weighing 120 ± 15 g were obtained from the Animal House of Dubai Pharmacy College for Girls, Dubai, UAE. Animals were housed in standard ventilated cages and were exposed to a 12-h light: 12-h dark cycle at 23 °C ± 2 °C. Animals had free access to rats’ pellets and water ad libitum. Animals were acclimatized for 14 days before the start of the experiment.

### 2.4. Study Design

Thirty-six male Wistar rats were divided into six groups at random. This includes a control group receiving distilled water, and five experimental groups (MSG, Maca, Fenugreek, Spirulina and Tribulus) receiving 900 mg/kg/day of MSG dissolved in water for 45 days. Subsequently, the animals in the experimental groups were administered 500 mg/kg/day of the respective plant extract via intragastric oral gavage for an additional 35 days, while the MSG group received distilled water only (Table 1).

After the treatment period, animals were euthanized using sevoflurane inhalation; subsequently, the testes and epididymis, seminal vesicles, and prostate glands were harvested and weighed.

#### 2.4.1. Sperm Retrieval for Motility, Concentration, and Morphology

The left epididymis was defatted after harvesting and placed in a petri dish containing 2 mL of DMEM-Hams F-12 nutrient media (Sigma Chemicals, St. Louis, MO, USA) at 37 °C. Following rinsing, sperm for motility analysis was collected by dissecting the caudal region and allowing spermatozoa to swim out for 30 s [34]. For concentration and morphology analysis, the caudal area was further dissected into smaller pieces and left for 5 mins allowing a maximum number of spermatozoa to swim out. The pieces were removed after 5 mins, and the sperm solution was mixed until homogenous. From the 2 mL solution, 10 µL was diluted in 50 µL of DMEM-Hams. Subsequently, 2 µL of this solution was placed in a chamber slide and analyzed using Computer-aided Sperm Analysis (CASA) with a Sperm Class Analyzer (SCA, Microptic, Spain). The dilution was performed to prevent cell overlap and facilitate accurate concentration measurement by the SCA in the sperm solution. Morphological analysis followed established protocols, recording the percentage of morphologically normal spermatozoa [34].

#### 2.4.2. Hormone Analysis

Serum testosterone, FSH and LH concentrations were measured using ELISA kits from Elabscience^®^ (Elabscience, Houston, TX, USA). Assays were conducted according to the manufacturer’s instructions. The catalogue numbers for the hormones include testosterone (E-OSEL-R0003); FSH (E-EL-R0391); and LH (E-EL-R0026).

#### 2.4.3. Histomorphometric Evaluation

The right testes from both the control and experimental groups were immersed in a 10% formalin solution for fixation. Tissues remained in formalin for at least 48 h to ensure thorough fixation. Subsequently, the fixed tissues underwent routine histological processing, including standard staining with haematoxylin and eosin (H&E). The prepared histology slides were digitized using an Olympus DP80 digital camera attached to an Olympus BX63 automated fluorescence microscope (BX63, Olympus Life Science, Waltham, MA, USA).

All virtual slides were downloaded as stack folders comprising EST file extensions. An EST file is a data file that contains the actual full-resolution image data. The EST files were then exported to the open-source software, QuPath version 0.4.3, for whole slide image analysis at 100× and 200× magnification [35].

**Figure 1:**
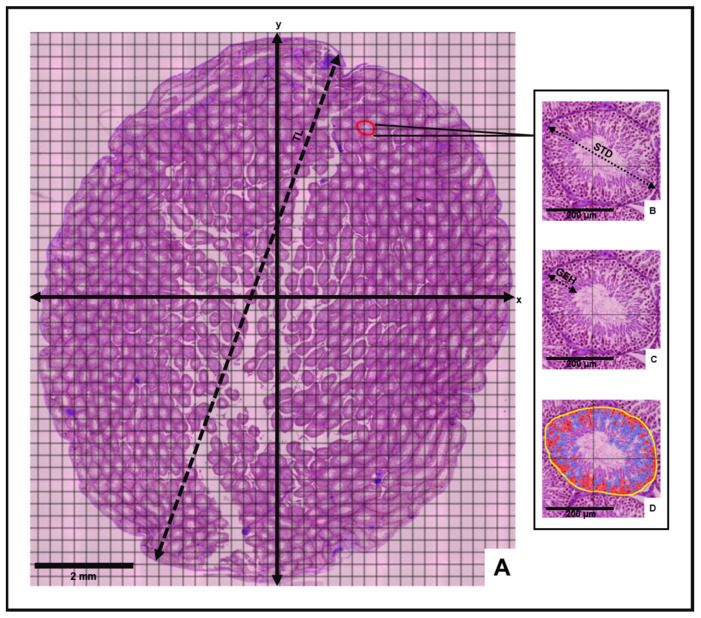
Histomorphometric evaluation of the testis. (**A**) Cross-sectional view of testis depicting measurement of testis length (TL), vertical and horizontal axes (x and y) and four histological fields. Insets: Measurement of (**B**) seminiferous tubule diameter (STD), (**C**) germinal epithelium height (GEH) and **(D**) number of cells in seminiferous tubules (Yellow = seminiferous tubule perimeter, Red = acidophilic cells, Blue = Basophilic cells) on selected tubular cross-section.

Morphometric and stereological analyses of the testes were then conducted to determine mean histological parameters for the control and experimental animal groups (Figure 1). Following the incorporation of a counting grid on each slide, vertical (y) and horizontal (x) axes were inserted to divide the grid into equidistant histological field quadrants (Figure 1). The mean testicular length (TL) was measured from an average of three line annotations extending between opposite poles of the long axis of the cross-sectional testis. Three circular tubular cross sections were randomly selected in each quadrant to determine mean values for the seminiferous tubule diameter (STD), germinal epithelium height (GEH) and number of cells in seminiferous tubules (NCST). The mean STD was measured between opposite poles of the outermost tunica propria layer across the minor and major axes of the tubule according to Tripathi et al. [36]. The mean GEH was measured from the basal membrane of the seminiferous tubule to the tubular lumen [37].

Best-fit polygon annotations were applied to the selected tubular cross-sections in each quadrant. Through the “cell detection” command of the “Analyze” tool, the number of cells in the selected tubular cross section was generated. Mean morphometric values for each slide were estimated by averaging the sum of the measurements obtained in each quadrant. Morphological observation of all virtual slides was also performed.

#### 2.4.4. Statistics

GraphPad Prism™ software (GraphPad™ Software, Version 10.2.1, San Diego, CA, USA) was used for the statistical analysis. Normal data distribution was measured using the Shapiro–Wilk, and Kolmogorov–Smirnov normality tests. When data passed normality tests, a one-way ANOVA of variance with a Tukey’s post hoc test was performed. Where data were not evenly distributed, a Kruskal–Wallis test and a Dunn’s post hoc test were performed. A probability level of *p* < 0.05 was considered statistically significant and results are expressed as mean ± SD.

## 3. Results

### 3.1. Phytochemical Profiling of the Plant Extracts

#### 3.1.1. Yield of Plant Extracts

The yields of the different extracts were as follows; *Lepidium meyenii*, *Trigonella foenum-graecum*, *Tribulus arabica*, and *Spirulina platensis*, 88, 36.2, 27, and 25.3 g, respectively. The percentage yields were calculated and were represented as 8.8%, 3.62%, 2.7%, and 2.53%, respectively.

*Lepidium meyenii* exhibited the highest yield (8.8%) among all the plants studied.

#### 3.1.2. Total Phenolic and Flavonoid Contents

As shown in Table 2, *Trigonella foenum-graecum* exhibited the highest phenolic content (1.538%), followed by *Spirulina platensis* (0.808%) and *Lepidium meyenii* (0.673%). The order of phenolic content from highest to lowest is as follows: *Trigonella foenum-graecum* > *Spirulina platensis* > *Lepidium meyenii* > *Tribulus arabica*.

On the other hand, *Spirulina platensis* exhibited the highest flavonoid content (0.22%), followed by *Tribulus arabica* (0.12%). The order of the flavonoid content from highest to lowest is as follows: *Spirulina platensis* > *Tribulus arabica* > *Trigonella foenum-graecum* > *Lepidium meyeniiMaca*.

#### 3.1.3. Quantitative Determination of the Phenolic Acids and Flavonoid Constituents by R-HPLC

**Figure 2:**
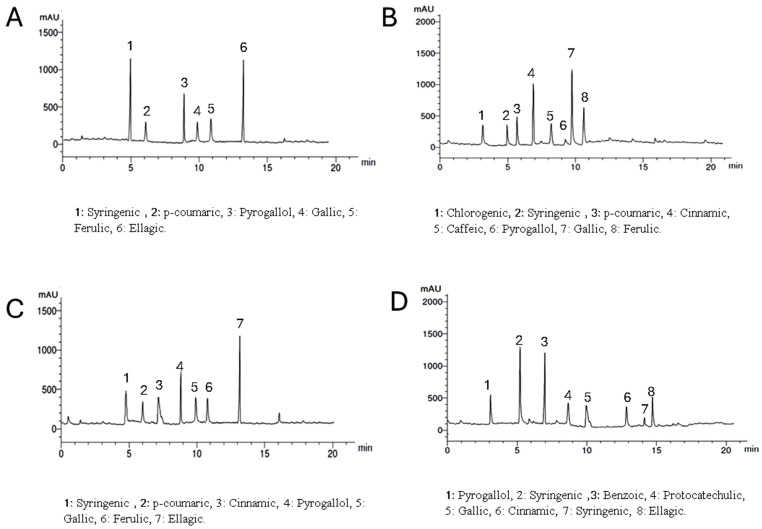
HPLC analysis of the phenolic constituents of the plants extract at wavelength 250 nm where, (**A**) is *Lepidium meyenii*, (**B**) is *Trigonella foenum-graecum*, (**C**) is *Tribulus arabica*, and (**D**) is *Spirulina platensis*.

Setting the detector at wavelength 250 nm allowed the identification of 6, 8, 7, and 7 components with the total percentages 3.907, 4.328, 4.466, and 3.817% for *Lepidium meyenii*, *Trigonella foenum-graecum*, *Tribulus arabica* and *Spirulina platensis*, respectively. Syringic acid, pyrogallol, and gallic acid were detected in all plants under investigation in different concentrations while chlorogenic acid was detected only in *Trigonella foenum-graecum*. Syringic acid and ellagic acid are the predominant phenolic acids in *Lepidium meyenii* (1.330% and 1.268%, respectively). On the other hand, gallic acid, and cinnamic acid are the predominant constituents in *Trigonella foenum-graecum* (1.574% and 1.308%, respectively). Meanwhile ellagic acid and pyrogallol are the major phenolics in *Tribulus arabica* (1.576 and 1.08%). Syringic acid was the major acid found in *Spirulina platensis* (1.263%) (Table 3 and Figure 2).

**Figure 3:**
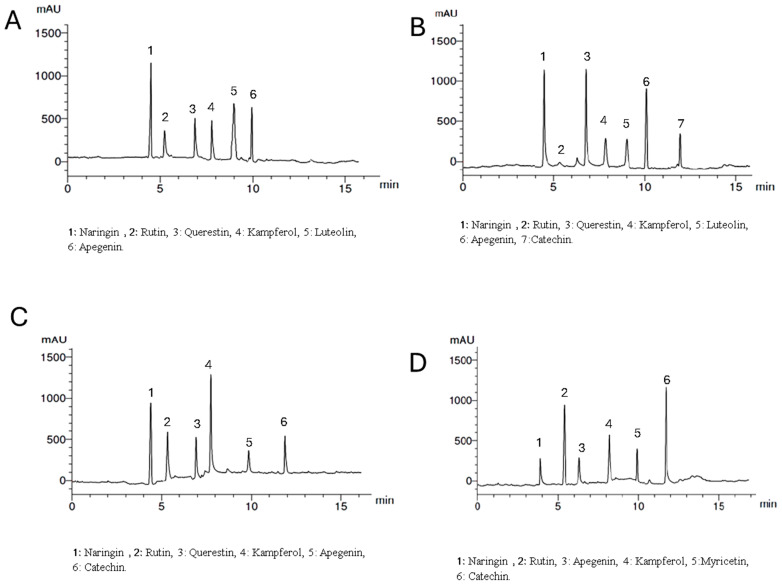
HPLC analysis of the flavonoid constituents of the plants extract at wavelength 360 nm where, (**A**) is *Lepidium meyenii*, (**B**) is *Trigonella foenum-graecum*, (**C**) is *Tribulus arabica*, and (**D**) is *Spirulina platensis*.

Setting the detector at wavelength 360 nm allowed the identification of 6, 7, 6, and 6 components with the total percentages 4.312, 5.314, 4.318, and 5.347% for *Lepidium meyenii*, *Trigonella foenum-graecum*, *Tribulus arabica*, and *Spirulina platensis*, respectively. All the identified flavonoid components were aglycone except rutin, which is a glycoside. Naringin, rutin, kaempferol, and apigenin were detected in all plants under investigation in different concentrations while myricetin was detected only in *Spirulina platensis*. Naringin is the predominant flavonoid aglycone in *Lepidium meyenii* (1.762%). On the other hand, quercetin, naringin and apigenin are the predominant constituents in *Trigonella foenum-graecum* (1.423, 1.263, and 1.126%, respectively). Meanwhile, naringin and Kaempferol are the major flavonoid aglycone in *Tribulus arabica* (1.236 and 1.021%). Catechin and rutin were the major found in *Spirulina platensis* (1.963 and 1.526%, respectively) (Table 4 and Figure 3).

#### 3.1.4. Antioxidant Activities of the Different Plants

The antioxidant activities of the different plant extracts were investigated. *Lepidium meyenii* exhibited the highest antioxidant activity, with a free radical inhibition of 63%, followed by *Trigonella foenum-graecum* at 50%, *Tribulus arabica at* 30%, and *Spirulina platensis* 25%, compared to the standard ascorbic acid 90%. The order of free radical inhibition percentages is as follows: *Lepidium meyenii* > *Trigonella foenum-graecum* > *Tribulus arabica* > *Spirulina platensis*.

### 3.2. The Effects of Maca, Fenugreek, Spirulina, and Tribulus on the Biometric Parameters of MSG-Treated Animals

After administration of MSG, a notable increase in body weight was observed compared to the control group. This pattern persisted across all experimental groups, even following intervention with the different plant extracts (Table 5), whereas no significant difference was observed in the testicular, epididymal, and seminal vesicle weights between the groups. However, there was a significant increase in the prostate gland in animals treated with *Tribulus arabica* compared to *Spirulina platensis* (0.5740 ± 0.1857 g vs. 0.3040 ± 0.1335 g; *p* = 0.028).

### 3.3. The Effects of Maca, Fenugreek, Spirulina, and Tribulus on the Sperm Parameters of MSG-Treated Animals

**Figure 4:**
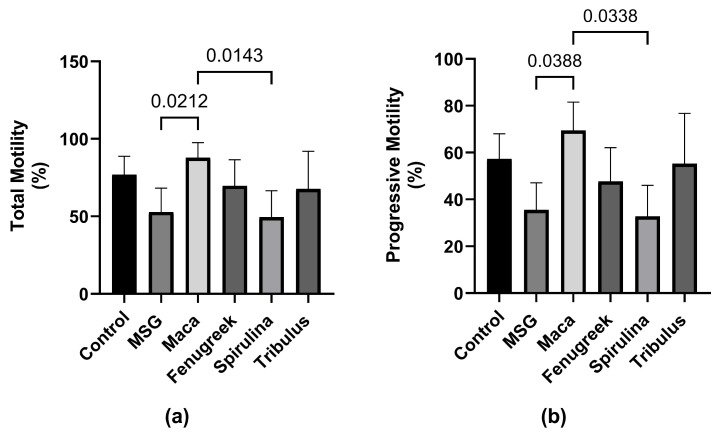
The effects of Maca, Fenugreek, Spirulina, and Tribulus on sperm motility of MSG-treated animals. (**a**) Total motility; (**b**) progressive motility. N = 4–6 per group. Figures show that MSG negatively affects sperm motility. After *Lepidium meyenii* supplementation, motility (total and progressive) was restored to values comparable to that of the control group. Thus, *Lepidium meyenii* was able to ameliorate the adverse effect of MSG on sperm motility.

Following exposure to MSG, there was a percentage decrease in total motility (−31.3%) of spermatozoa compared to the control group (Figure 4), which was restored upon treatment with Maca (*p* < 0.05). Additionally, there was a percentage increase in the total motility of animals that received Fenugreek and Tribulus compared to the MSG group.

**Figure 5:**
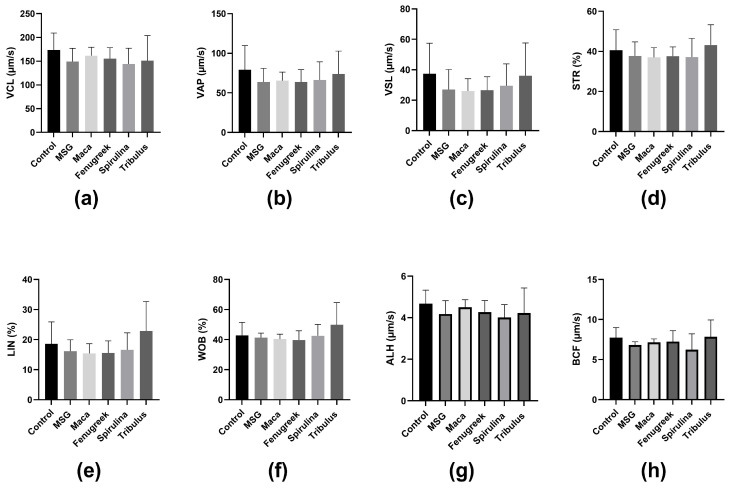
The effects of Maca, Fenugreek, Spirulina, and Tribulus on sperm kinematic parameters of MSG-treated animals. (**a**) Curvilinear Velocity (VCL); (**b**) average path velocity (VAP); (**c**) straight line velocity (VSL); (**d**) straightness (STR); (**e**) linearity (LIN); (**f**) wobble (WOB); (**g**) amplitude of lateral head displacement (ALH); (**h**) beat cross frequency (BCF). N = 4–6.

Similarly, there was a percentage reduction (−37.9%) in progressive motility of the animals that were administered MSG only compared to the control, whereas there was a significant increase in progressive motility of animals that received Maca compared to the MSG group (69.44 ± 12.12 versus 35.52 ± 11.59; *p* = 0.03) (Figure 4b). Nevertheless, there was no statistically significant difference in kinematic parameters between the different groups (Figure 5).

**Figure 6:**
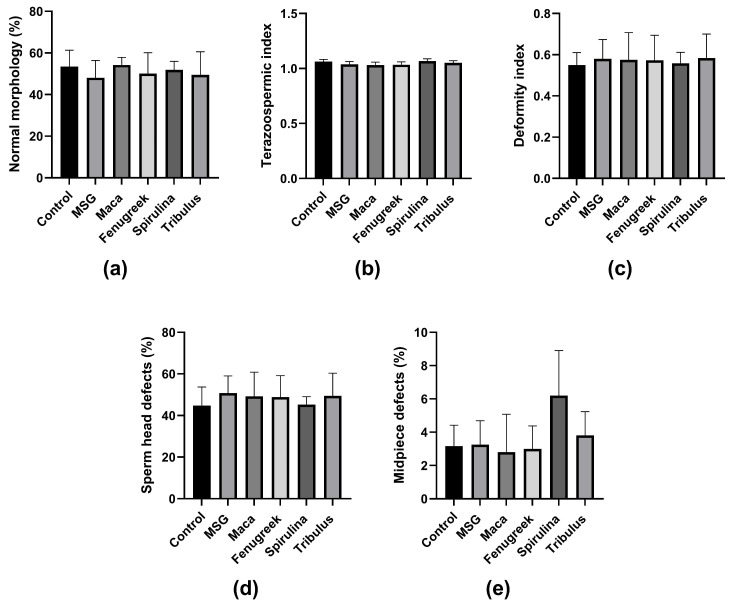
The effects of Maca, Fenugreek, Spirulina, and Tribulus on sperm morphology and morphometric parameters of MSG-treated animals. (**a**) Percentage of spermatozoa with normal morphology; (**b**) teratozoospermic index; (**c**) deformity index; (**d**) percentage of sperm head defects; (**e**) percentage of midpiece defects. N = 4–6.

Furthermore, there was no significant difference in the percentage of morphologically normal spermatozoa and other morphometric parameters between the groups (Figure 6a–e). Noteworthy, animals in the *Spirulina* group displayed the highest midpiece defects, which could be related to lower mitochondrial activity, and thus affect motility.

### 3.4. The Effects of Maca, Fenugreek, Spirulina, and Tribulus on Hormone Levels of MSG-Treated Animals

**Figure 7:**
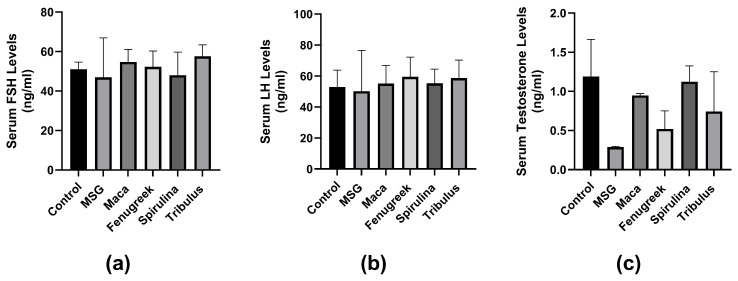
The effects of Maca, Fenugreek, Spirulina, and Tribulus on gonadal hormone levels of MSG-treated animals. (**a**) Serum follicle-stimulating hormone (FSH) levels; (**b**) serum luteinizing hormone (LH) levels; (**c**) serum testosterone levels. Treatment with MSG drastically lowered serum testosterone levels, which was percentage-wise increased after intervening with *Lepidium meyenii*, *Spirulina platensis*, *Tribulus arabica*, and *Trigonella foenum-graecum*.

There was no significant difference in serum FSH concentration between the groups (*p* = 0.6). However, there was a percentage decrease (−8%) in FSH concentration in animals treated with MSG only compared to control. Treatment with all infusions improved FSH levels, although not significant (Figure 7a). Similarly, there was no significant difference in LH concentration between the groups. Although there was a percentage decrease (−4.9%) in the MSG group compared to the control group. LH concentration was improved by all infusions percentage-wise (Figure 7b).

Additionally, there was no significant difference in serum testosterone concentration between the groups (*p* = 0.057). However, there was a percentage decrease (−76.5%) in testosterone levels of the MSG-treated group compared to the control group. Serum testosterone levels were improved in all infusion groups compared to the MSG group (Figure 7c).

### 3.5. The Effects of Maca, Fenugreek, Spirulina, and Tribulus on Testicular Morphology and Morphometrics of MSG-Treated Animals

**Figure 8:**
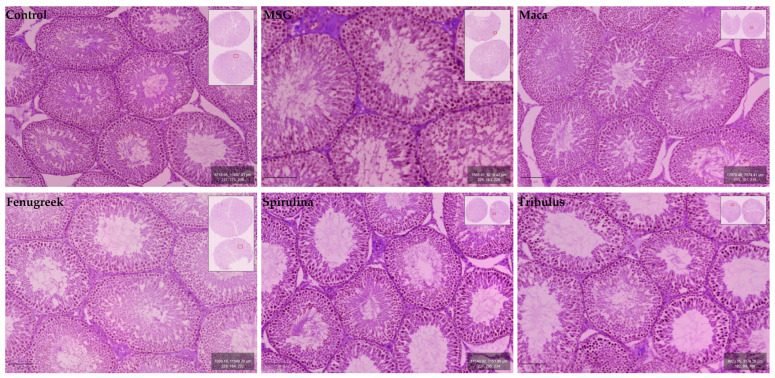
Micrographs showing histomorphological features of the cross-sectional seminiferous tubules in testes of control and experimental groups.

Testicular morphology was observed to be normal for the experimental animal groups, with all stages of spermatogenesis (i.e., spermatogonia, primary spermatocytes (pachytene), secondary spermatocytes, early spermatids, late spermatids, and spermatozoa) evident within the germinal epithelium of seminiferous tubules. The epithelial lining of seminiferous tubules exhibited the usual ordered stratified architecture with the presence of normal and healthy Sertoli cells. Many normal Leydig cells were also located within the interstitium of the testis (Figure 8).

As reflected in Table 6, the Fenugreek (*Trigonella foenum-graecum*) group presented with the highest mean TL and GEH. The latter parameter appeared to be marginally lower in the Maca (*Lepidium meyenii*) group, which incidentally also had the highest NCST. The mean STD was notably the largest in the MSG + H20 group. Although the mean STD, GEH and NCST were lowest in the control group, the MSG + H20 group presented with the shortest TL. As reflected in Table 6, the Fenugreek (*Trigonella foenum-graecum*) group presented with the highest mean TL and GEH. The latter parameter appeared to be marginally lower in the Maca (*Lepidium meyenii*) group, which incidentally also had the highest NCST. The mean STD was notably the largest in the MSG + H20 group. Although the mean STD, GEH and NCST were lowest in the control group, the MSG + H20 group presented with the shortest TL.

## 4. Discussion

Male infertility is becoming a global health problem, and it is attributable to more than 50% of couples’ infertility. Various factors have been linked to subfertility, encompassing inadequate dietary intake, adverse effects of medications, pathological changes or disease states, and intoxication, which may involve excessive consumption of MSG. Although various conventional treatment strategies are available for treating subfertility, these methods are sometimes too expensive, invasive, and cumbersome. In the search for alternative treatment methods with minimal invasion and side effects, the use of natural herbs and plants has gained more traction [38]. Thus, the present study evaluated the potential effect of natural herbs, including *Lepidium meyenii*, *Trigonella foenum-graecum*, *Tribulus arabica*, and *Spirulina platensis* in treating male reproductive parameters impairment in MSG-induced reproductive dysfunctions in male rats in.

This current study revealed that *Lepidium meyenii*, *Trigonella foenum-graecum*, and *Tribulus arabica*, respectively, have potential benefits for male fertility with the most significant results seen with *Lepidium meyenii*. These plants offer various constituents that can contribute to male reproductive health and fertility. *Lepidium meyenii* exhibited the highest antioxidant activity, with a free radical inhibition of 63% which may reduce oxidative stress (OS) and improve sperm quality. *Trigonella foenum-graecum*, which has been shown to have phyto-estrogenic effects, provides nutrients and helps regulate blood sugar levels [39], exhibited a free radical inhibition of 50%. Although *Tribulus arabica* exhibited a free radical inhibition of 30%, and *Spirulina platensis* showed a 25% free radical inhibition.

Following MSG administration, animals displayed reduced sperm motility (total and progressive), which is consistent with findings from other studies [40]. On the other hand, treatment with *Lepidium meyenii* ameliorated the adverse effects of MSG by restoring sperm total motility and progressive motility to levels comparable to the control group. This is in line with several studies that reported the positive effects of *Lepidium meyenii* on sperm quality, including sperm count, motility, and morphology [41,42]. This ameliorative effect may be attributed to its antioxidant activity, which can help reduce OS. Although reactive oxygen species (ROS) levels were not assessed in this study, it is widely documented that oxidative stress (OS) detrimentally affects sperm motility through elevated ROS generation. The sperm plasma membrane has a high percentage of polyunsaturated fatty acids [43] which are essential for sperm motility. However, these fatty acids are susceptible to ROS, leading to lipid peroxidation and subsequent membrane invasion [44]. Lipid peroxidation takes place when reactive oxygen species (ROS) interact with fatty acid chains, resulting in the formation of lipid peroxyl radicals. These peroxyl radicals then further react with fatty acids, generating additional ROS. This cascade of free radical reactions results in lipid breakdown. However, studies have shown that protecting against OS-induced damage requires addressing the underlying cause [45] and subsequently suppressing pro-oxidants with antioxidants [46]. Thus, it is suggested that the improved sperm motility observed following treatment with *Lepidium meyenii* may be partly attributed to its antioxidant activity, which could mitigate the stress induced by excessive MSG consumption. Invariably, the antioxidant properties of *Lepidium meyenii* may help protect spermatozoa from oxidative damage and improve overall fertility. Maca’s secondary metabolites, such as macamides and macaenes, along with other lipid-soluble components, may influence the reproductive system by altering the antioxidant-oxidant balance, potentially improving semen quality possibly through the stimulation of glucosinolates. However, the relationship between macamides, macaenes, glucosinolates, and the medicinal effects of *Lepidium meyenii* on male gonads remains unclear due to insufficient data. Further research is needed to explore the specific macamides and glucosinolates unique to *Lepidium meyenii* particularly their role in activating androgen signaling based on their distinct structures. Additionally, a study on mice revealed that Maca could counteract the effects of ketoconazole, which inhibits the synthesis of adrenal steroids and testosterone by blocking the P-450 enzyme system. By restoring cytochrome P450 functionality, *Lepidium meyenii* appears to enhance sperm motility, suggesting its potential to reverse chemical subfertility related to cytochrome P450 and androgen production. By restoring cytochrome P450 functionality, Maca appears to enhance sperm motility, suggesting its potential to reverse chemical subfertility related to cytochrome P450 and androgen production [47].

Notably, *Trigonella foenum-graecum* has been utilized to promote reproductive well-being and address male infertility concerns [39]. Several factors contribute to the potential benefits of *Trigonella foenum-graecum* for fertility, including its phytoestrogenic properties [48]. *Trigonella foenum-graecum* encompasses constituents such as trigonellin, which have undergone scrutiny for their phytoestrogenic attributes. Phytoestrogens are plant-derived compounds capable of binding to estrogen receptors in the body and eliciting estrogen-like effects. These phytoestrogens are believed to aid in hormonal regulation and support reproductive health [49]. Furthermore, *Trigonella foenum-graecum* boasts a significant nutritional profile, serving as a source of various nutrients, vitamins, minerals, and antioxidants [50]. The present study demonstrated substantial antioxidant activity, evidenced by a 50% inhibition of free radicals. These nutrients and antioxidants play a vital role in overall health and contribute to the optimal functioning of the reproductive system [39]. Additionally, *Trigonella foenum-graecum* has been examined for its potential antidiabetic effects, primarily attributed to the presence of 4-hydroxyisoleucine. Maintaining stable blood sugar levels is crucial for reproductive health, as imbalances in blood sugar can adversely impact fertility [51].

Of interest, *Tribulus arabica* has potential benefits for male fertility. It may regulate hormone levels, including testosterone, which is important for reproductive function [52]. Moreover, it may also improve sperm health, motility, and morphology, contributing to successful conception [51]. Its antioxidant properties can protect spermatozoa from oxidative stress [53]. The present study showed that Tribulus exhibited an antioxidant activity with a free radical inhibition of 30%. Additionally, Tribulus may promote nitric oxide production, improving blood flow to reproductive organs [54]. It is also known as a natural aphrodisiac, potentially enhancing libido and sexual function, indirectly supporting fertility [55].

It is noteworthy to mention that myristic acid has been identified in three of these plants, including *Lepidium meyenii, Tribulus arabica*, and *Trigonella foenum-graecum*, which have demonstrated potential anti-infertility effects. Additionally, a study indicated that the administration of myristic acid offers a promising therapeutic strategy for protecting the testes against oxidative stress and inflammation induced by hyperglycemia. This compound exhibits efficacy in preserving reproductive function and enhancing fertility in males with diabetes [56].

The presence of myristic acid, a fatty substance, has been found to be crucial in enhancing sperm motility and acrosome reaction (AR). Notably, a study observed a minor decrease in the levels of saturated fatty acids, along with a significant decrease in monounsaturated and polyunsaturated fatty acids within the phospholipid fraction, which is a primary constituent of sperm membranes. This alteration significantly influences the quality of semen, wherein these fatty acids play a pivotal role. Analyzing the quantity and quality of fatty acids present in sperm lipid composition can potentially provide an explanatory framework for unexplained infertility. Polyunsaturated fatty acids play a significant role in sperm motility and the ability to penetrate the egg [57,58,59].

Of note, *Tribulus arabica*, *Trigonella foenum-graecum*, and *Lepidium meyenii* in the current study demonstrated the presence of total flavonoid in a percentage of 0.12%, 0.08%, and 0.01%, respectively (Table 2). Additionally, HPLC analysis of these three plants detected rutin, apigenin, quercetin, syringic acid, and ferulic acid (Figure 2 and Figure 3). The combination of these flavonoids and phenolic acids exhibits synergistic activity, as they all possess antioxidant and anti-inflammatory properties. These compounds are believed to combat oxidative stress, which can damage sperm DNA and impair sperm function. Therefore, this combination may potentially improve sperm quality and reduce inflammation in the testes [60].

Furthermore, flavonoids have been reported to improve male reproductive system dysfunction, such as testicular structural disruption and spermatogenesis disturbance, owing to their antioxidant, anti-inflammatory, immunity-boosting, anti-apoptotic, anticarcinogenic, anti-allergic, and antiviral properties. In a study where spermatogenesis was inhibited due to testicular weight loss and structural disruption, which led to a decline in sperm quantity and quality, a flavonoid supplement of 20 mg/kg significantly enhanced testicular histology and reduced germ cell apoptosis by reversing the vacuolation of the germinal epithelium, separation of germ cells from the basal lamina, and shedding of immature germ cells [61].

In the present study, the histological features of the testis reflected normal morphology and were not affected by the administration of phytochemical constituents to MSG-induced Wistar rats. Although exposure to MSG causes histological alternations to the interstitium of the testis and the germinal epithelium and lumina of seminiferous tubules, this is in contrast to our observations [62,63] (rat male reproductive system (NF-kB) levels in response to monosodium glutamate; environmental factor). However, our findings are in agreeance with Abd-Elkareem et al. [64] and Baradaran et al. [65]. Histopathological and biochemical effect of quercetin on monosodium glutamate supplementation-induced testicular toxicity [66], both of whom reported that the addition of phytochemical agents *Nigella sativa* L. seeds and Quercetin, respectively, induced visible transition of the seminiferous tubule epithelium to normal in the initial MSG-treated groups. It may be postulated that a similar reparative process occurred with the phytochemical agents in the present study. It is noteworthy that our study is the first to describe the architecture of the seminiferous tubule epithelium in relation to the combined administration of multiple phytochemicals in MSG-treated Wistar rats.

Although previous studies highlighted the individual alleviative role played by phytochemical agents such as *Spirulina*, Quercetin flavonoid and *Nigella sativa* L. seeds on reproductive function, none have incorporated a combination of phytochemicals into their investigations [64,66,67]. Moreover, very few have shed light on the reparative effect of these phytochemicals on histomorphometric parameters in MSG-induced animals. This applies specifically to the TL and NCST of the present study as no comparative mean values exist. On the contrary, our mean STD and GEH values were higher than studies exploring the effects of *Tribulus* and *Nigella sativa* L. seeds [64,68]. Statistically significant differences in mean STD were yielded for the control vs. MSG + H20 and MSG + H20 vs. *Tribulus* comparisons which may be indicative of the markedly higher mean STD noted in the MSG + H20 group of our study. Although the STD is expected to increase from a control group to one that was treated with MSG, the aforementioned statistical significance does not explain the lower STD value obtained for the *Tribulus* group as it is known to exert a protective effect against testicular damage [69]. The mean GEH values were recorded to be greater in the Maca and Fenugreek groups of our study, but lower than that documented by Kianifard et al. [67] who concluded that MSG promotes gonadotoxic effects of the antimalarial drug, Quinine, on testicular tissue. This may be attributed to the cellular characteristics of testicular tissue which renders it vulnerable to factors that are unique to an environment [70]. Interestingly, a comparison of the mean GEH between control and Fenugreek animal groups resulted in a statistically significant difference which may be due to the increases seen when GEH magnitude was traced from the Control and MSG + H20 groups to the Fenugreek group. This improvement in histomorphometrics of the testis is suggestive of the protective effects afforded by Fenugreek. These results are also similar to Arafa et al. [71] who showed that the antioxidant, anti-inflammatory and antifibrotic effects of Fenugreek on cadmium-induced testicular injury and hepatic dysfunction make it a promising medicinal herb. With the exception of the STD parameter, treatment of the phytochemical agents diminished histomorphometric alternations of the TL, GEH, and NCST, which were caused by the initial administration of MSG.

Additionally, exposure to MSG reduced serum testosterone levels, while LH and FSH levels remained unchanged. Several studies have similarly reported decreased serum testosterone levels following MSG exposure [14,40]. However, findings on LH and FSH expression after MSG exposure remain controversial. For instance, Koohpeyma et al. reported a decrease in both serum LH and FSH following MSG administration [40], while Abd-Elkareem et al. reported an elevation in serum LH [64]. The discrepancies may be due to differences in the concentration of MSG administered. Nonetheless, studies have consistently reported a decrease in testosterone concentration, whether in serum or testicular tissue, after MSG administration.

However, treatment with *Lepidium meyenii*, *Spirulina platensis*, *Tribulus arabica*, and *Trigonella foenum-graecum* improved serum testosterone levels, although not significantly.

## 5. Conclusions

In conclusion, the current study findings demonstrate the potential benefits of *Lepidium meyenii*, *Trigonella foenum-graecum*, and *Tribulus arabica* for male fertility, with *Lepidium meyenii* showing the most significant results. It exhibits strong antioxidant activity, reducing oxidative stress and improving sperm quality. Moreover, *Lepidium meyenii* effectively counters the adverse effects of MSG on sperm motility. We thought that improving semen quality by Maca possibly through the stimulation of glucosinolates. However, the relationship between macamides, macaenes, glucosinolates, and the medicinal effects of *Lepidium meyenii* on male gonads remains unclear due to insufficient data. Further research is needed to explore the specific macamides and glucosinolates unique to *Lepidium meyenii* particularly their role in activating androgen signaling based on their distinct structures. Additionally, given MSG’s detrimental impact on male reproductive functions, this study emphasizes the importance of educating the public about its harmful effects and suggests considering alternatives or using it alongside antioxidants to mitigate its effects.

## Figures and Tables

**Table 1:** Experimental study design.

Groups	Intervention	Treatments
Control	DW	DW
MSG	900 mg/kg/day	DW
as *Lepidium meyenii*	900 mg/kg/day	500 mg/kg/day
*Trigonella foenum-graecum*	900 mg/kg/day	500 mg/kg/day
*Spirulina platensis*	900 mg/kg/day	500 mg/kg/day
*Tribulus arabica*	900 mg/kg/day	500 mg/kg/day

Treatments and interventions were administered via intragastric oral gavage. DW = distilled water, MSG = Monosodium glutamate.

**Table 2:** Percentage of total phenolic and flavonoid contents.

Name of Plant	Percentage of Phenolic (%) *	Percentage of Flavonoids (%)
*Lepidium meyenii* (Maca)	0.673%	0.010%
*Trigonella foenum-graecum* (Fenugreek)	1.538%	0.080%
*Tribulus arabica* (Tribulus)	0.074%	0.120%
*Spirulina platensis* (Spirulina)	0.808%	0.220%

* per 100 g dry plant material.

**Table 3:** HPLC analysis of the phenolic constituents of the plant extract at wavelength 250 nm.

Phenolic Content	RT	*Lepidium meyenii*	*Trigonella foenum-graecum*	*Tribulus arabica*	*Spirulina platensis*
Chlorogenic acid	3.11	-	0.311	-	-
Syringic acid	5.00	1.330	0.212	0.512	1.263
p-Coumaric acid	6.00	0.214	0.326	0.256	-
Benzoic acid	6.90	-	-	-	0.957
Cinnamic acid	7.00	-	1.308	0.416	0.154
Caffeic acid	8.00	-	0.211	-	-
Protocatechulic	8.70	-	-	-	0.232
Pyrogallol	9.20	0.552	0.056	1.08	0.374
Gallic acid	9.80	0.214	1.574	0.269	0.405
Ferulic acid	11.00	0.329	0.656	0.357	-
Ellagic acid	13.00	1.268	-	1.576	0.432
Total percentage		3.907	4.328	4.466	3.817
No. of identified components		6	8	7	7

**Table 4:** HPLC analysis of the flavonoid constituents of the plant extract at wavelength 360 nm.

Flavonoid Content	RT	*Lepidium meyenii*	*Trigonella foenum-graecum*	*Tribulus arabica*	*Spirulina platensis*
Naringin	4.6	1.762	1.263	1.236	0.233
Rutin	5.2	0.251	0.102	0.641	1.526
Quercetin	7.0	0.563	1.423	0.510	-
Kaempferol	7.9	0.474	0.427	1.021	0.719
Luteolin	9.0	0.726	0.399	-	-
Myricetin	9.9	-	-	-	0.584
Apigenin	10.0	0.536	1.126	0.147	0.322
Catechin	12.0	-	0.574	0.763	1.963
Total percentage		4.312	5.314	4.318	5.347
No. of identified components		6	7	6	6

**Table 5:** Body and organ weights.

Group	Control	MSG	*Lepidium meyenii*	*Trigonella foenum-graecum*	*Spirulina platensis*	*Tribulus arabica*
Body weight (g)	258.88 ± 41.14	324.32 ± 22.87	308.08 ± 8.00	354.22 ± 55.04 *	315.20 ± 19.58	350.92 ± 60.26 *
Testis (g)	1.55 ± 0.20	1.5 ± 0.13	1.56 ± 0.15	1.6 ± 0.18	1.6 ± 0.17	1.6 ± 0.24
Epididymis (g)	0.5 ± 0.01	0.54 ± 0.07	0.53 ± 0.06	0.57 ± 0.07	0.58 ± 0.08	0.56 ± 0.09
Prostate (g)	0.29 ± 0.09	0.44 ± 0.8	0.46 ± 0.89	0.45 ± 0.14	0.30 ± 0.13	0.57 ± 0.19 ^†^
Seminal vesicles (g)	1.06 ± 0.7	1.34 ± 0.27	1.47 ± 0.25	1.53 ± 0.41	1.50 ± 0.33	1.51 ± 0.39

* *p* < 0.05 versus control, ^†^ *p* < 0.05 versus Spirulina, N = 4–6 per group.

**Table 6:** Histomorphometric parameters of testicular tissue in the control and experimental animal groups.

Animal Group	Testis Length (TL) (μm)	Seminiferous Tubule Diameter (STD) (μm)	Germinal Epithelium Height (GEH) (μm)	Number of Cells in Seminiferous Tubules (NCST)
Control	10,098.27 ± 633.01	410.44 ± 57.86 *	74.55 ± 17.85 **	374.13 ± 237.16
MSG + H_2_0	9798.77 ± 1071.27	485.93 ± 73.62 *#	80.32 ± 8.92	471.33 ± 168.56
Maca	10,353.16 ± 659.38	474.70 ± 72.69	84.61 ± 8.01	488.4 ± 254.40
Fenugreek	10,433.32 ± 361.84	476.47 ± 62.9	84.80 ± 9.57 **	477.9 ± 248.26
Spirulina	10,125.79 ± 288.73	455.76 ± 100.75	79.04 ± 9.27	446.00 ± 208.79
Tribulus	10,074.87 ± 1203.49	418.04 ± 74.73 #	77.28 ± 8.13	376.65 ± 172.65

*p* < 0.05, * Control vs. MSG + H20, ** Control vs. Fenugreek, # MSG + H20 vs. Tribulus.

## Data Availability

Data are contained within the article.
